# Crystal Structure of an EAL Domain in Complex with Reaction Product 5′-pGpG

**DOI:** 10.1371/journal.pone.0052424

**Published:** 2012-12-20

**Authors:** Julien Robert-Paganin, Sylvie Nonin-Lecomte, Stéphane Réty

**Affiliations:** 1 Laboratoire de Cristallographie et RMN biologiques, UMR 8015-Cente National de la Recherche Scientifique, Paris, France; 2 Faculté de Pharmacie, Université Paris Descartes, Paris, France; University of Canterbury, New Zealand

## Abstract

FimX is a large multidomain protein containing an EAL domain and involved in twitching motility in *Pseudomonas aeruginosa*. We present here two crystallographic structures of the EAL domain of FimX (residues 438–686): one of the apo form and the other of a complex with 5′-pGpG, the reaction product of the hydrolysis of c-di-GMP. In both crystal forms, the EAL domains form a dimer delimiting a large cavity encompassing the catalytic pockets. The ligand is trapped in this cavity by its sugar phosphate moiety. We confirmed by NMR that the guanine bases are not involved in the interaction in solution. We solved here the first structure of an EAL domain bound to the reaction product 5′-pGpG. Though isolated FimX EAL domain has a very low catalytic activity, which would not be significant compared to other catalytic EAL domains, the structure with the product of the reaction can provides some hints in the mechanism of hydrolysis of the c-di-GMP by EAL domains.

## Introduction

Cyclic di-GMP (bis-(3′-5′) cyclic dimeric guanosine monophosphate, c-di-GMP) was first considered as an allosteric regulator of cellulose production in *Gluconoacetobacter xylinus* [1], [2]. Further studies revealed that c-di-GMP is a second messenger exclusively present in Eubacteria and involved in the regulation of various essential biological processes like twitching motility, swarming, quorum sensing, virulence and surface adhesion [3], [4], [5]. In several bacteria, the switch between planktonic and biofilm formation is controlled by the concentration of c-di-GMP. The increase in intracellular c-di-GMP concentration leads to biofilm formation while its decrease enhances bacterial mobility and virulence [6]. The targets of c-di-GMP are either proteins with a PilZ domain [7] or RNA riboswitches [8].

Three protein domains, GGDEF, EAL, and HD-GYP, have been reported to be involved in c-di-GMP metabolism. They are named after the conserved amino-acids motifs in these domains [9]: GGDEF have diguanylate cyclase (DGC) activity and manage the synthesis of c-di-GMP. EAL domains exhibit phosphodiesterase A (PDE-A) activity and are responsible for the conversion of c-di-GMP into 5′-phosphoguanylyl-(3′-5′)-guanosine (5′-pGpG). HD-GYP domains also carry a phosphodiesterase activity, which can hydrolyze c-di-GMP into 5′-pGpG and further into two GMP molecules. Though it has been evidenced *in vitro* that EAL domains can pursue the hydrolysis of 5′-pGpG into two GMP molecules [10], this second reaction is however very slow compared to the reaction carried out by HD-GYP domains, which raises the question as to its occurrence *in vivo* [11]. Although most recent studies assume that DGC and PDE activities monitor the intra-cellular c-di-GMP concentration, the understanding of the complex molecular mechanisms regulating these activities and the signal transduction by c-di-GMP is very poor [12], [13].

Bacteria using c-di-GMP signaling networks produce several proteins containing an EAL domain. EAL domains have been reported to be catalytically active in presence of either Mg^2+^ or Mn^2+^. Conversely, the PDE-A activity is inhibited in presence of calcium ions. The comparison of active EAL domains and of degenerated non-catalytic EAL domains has allowed to identify 14 polar residues important for the PDE activity. Based on the structure of the EAL domain containing protein YkuI from *Bacillus subtilis* and site directed mutagenesis on the EAL domain containing protein RocR from *P. aeruginosa,* a mechanism explaining the phosphodiesterase activity by the EAL domains has been proposed [14]. It is an Mg^2+^ base-assisted catalysis, but the number of Mg^2+^ (one, two or three) necessary for catalysis remains elusive [15]. In a more recent study [16], 13 EAL domains from various organisms were purified and their activity was measured. Ten conserved residues important for the PDE-A activity were identified, all these residues being already defined as important in previous studies [14]. This, together with structural comparisons, leads the authors to conclude that the hydrolysis of c-di-GMP involves two Mg^2+^ ions.

FimX (PA4959) from *P. aeruginosa* is a 76,000 Da protein identified in mutants lacking twitching motility in response to environmental factors [17]. Twitching motility is a form of surface translocation mediated by extension, tethering and retraction of type IV pili, which are polar structures also required for biofilm formation and adherence. This lack of motility is reminiscent of an increase of c-di-GMP cellular concentration, and thus FimX is expected to down-regulate c-di-GMP hydrolysis. A FimX homolog from *Xanthomonas axonopodis*, a citrus pathogen, has been reported to interact with PilZ, a protein involved in type IV pilus biogenesis [18]. FimX from *P. aeruginosa* contains a CheY-like response regulator domain (REC), a PAS-PAC domain commonly involved in environmental sensing, and one GGDEF and one EAL domain. FimX is located at one pole of the bacterial cell probably by its N-terminal REC domain. The full-length protein possesses a PDE-A activity stimulated by Mg^2+^ and Mn^2+^ but strongly inhibited by Ca^2+^, consistently with what has been described for other proteins with an EAL domain [19]. The FimX GGDEF domain is degenerated and does not display any diguanylate cyclase activity, but may stimulate the PDE-A activity in presence of GTP. Such allosteric regulation of an active EAL domain by a degenerated and inactive GGDEF recalls that described for CC3396, another dual GGDEF-EAL domain protein from *Caulobacter crescentus* [20]. The isolated FimX EAL domain doesn’t show significant catalytic activity [19], [21]. A comparative bioinformatics approach showed that FimX-EAL domain lacks several essential residues for catalysis compared to active EAL domains from other proteins [14]. The crystallographic structures of the FimX EAL and GGDEF domains have been solved [21] and show that FimX-EAL can bind c-di-GMP despite the lack of some amino acids predicted to be important for the catalysis. However, neither DGC nor PDE activity was detected for the (full-length) FimX protein used in this study, which is not consistent with what was previously shown [19]. In the complex, c-di-GMP is bound through interactions with 5 of the 10 amino acids identified as essential (Tyr673, Glu654, Asp507, Arg479 and Leu477). Interestingly, the stœchiometry of FimX-EAL in the crystals depends on the presence the ligand. The apo state of FimX-EAL and the dual FimX-GGDEF-EAL crystallize as a dimer but FimX-EAL in complex with c-di-GMP crystallizes as a monomer. FimX-EAL monomers in the apo and complex states differ by few conformational changes. SAXS and analytical centrifugation data showed that FimX would dimerize through its N-terminal REC domain in solution [21].

Here we solved the crystallographic structure of FimX-EAL bound to 5′-pGpG and show that this domain doesn’t display major structural rearrangements whether bound to 5′-pGpG, c-di-GMP or in apo form. We also probe its affinity for c-di-GMP and 5′-pGpG in solution by NMR spectroscopy and determined which protons of the ligand are recognized by the protein.

## Results

### Overall Structures

**Figure 1:**
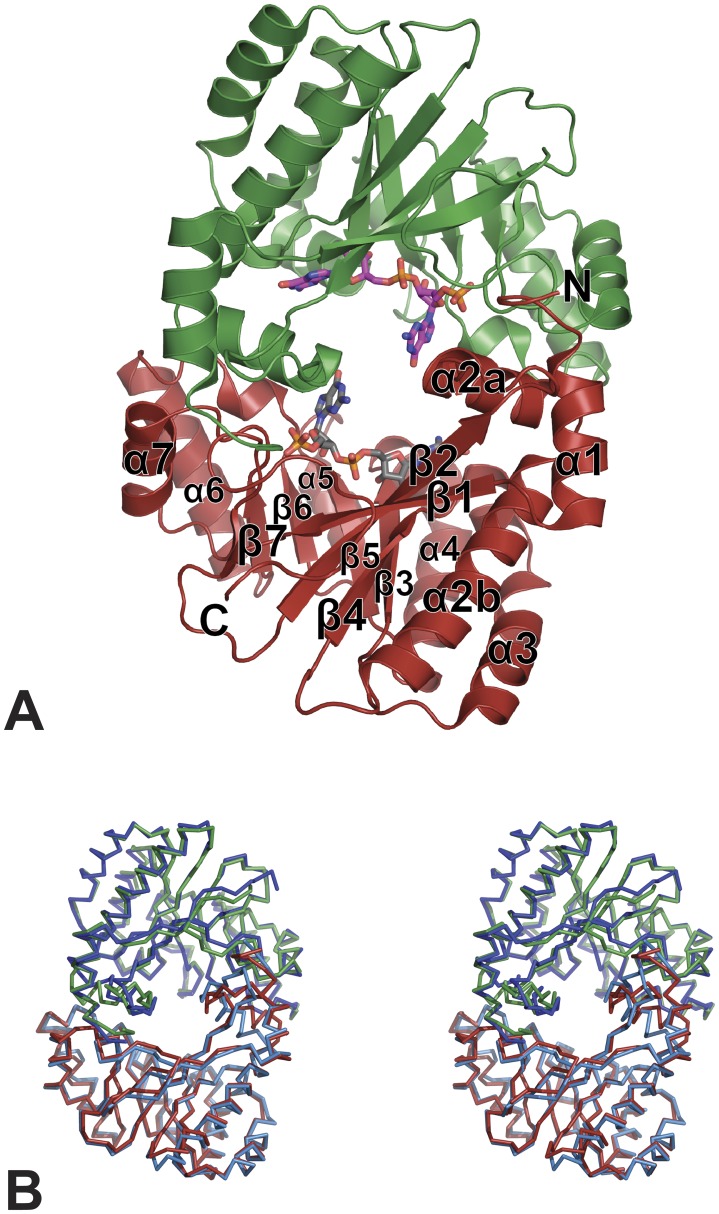
Structure of the homodimer of EAL domain of FimX with bound 5′-pGpG. (A) Structure of the homodimer of EAL domain of FimX with bound 5′-pGpG. The secondary structure elements are labelled on one monomer. (B) Stereo view of the superposition of homodimer of EAL domain of FimX in apo form crystallized in spacegroup P2_1_2_1_2_1_ (dark blue and light blue) on the homodimer of FimX EAL in complex with 5′-pGpG crystallized in spacegroup P4_3_2_1_2 (red and green).

The FimX-EAL domain (residues 438–686) was crystallized alone and in presence of the substrate c-di-GMP in space groups P2_1_2_1_2_1_ and P4_3_2_1_2 respectively. Crystals diffracted to 2.3 Å and 2 Å resolution respectively (Table 1). The structures were solved using molecular replacement using the EAL domain model tdEAL from *Thiobacillus denitrificans* (PDB code 2R6O) as search model. Both structures contain two molecules per asymmetric unit related by a non-crystallographic two-fold axis (Figure 1).

**Table 1:** Data Collection and Refinement Statistics.

Structure	FimX-EAL apo	FimX-EAL bound to 5′-pGpG native
Space Group	P2_1_2_1_2_1_	P4_3_2_1_2
Unit Cell	a = 64.530 Å b = 87.660 Å c = 108.240 Å α = β = γ = 90°	a = 104.33 Å, b = 104.33 Å, c = 154.24 Å α = β = γ = 90°
X-ray source	SOLEIL (PROXIMA 1)	SOLEIL (PROXIMA 1)
Wavelength (Å)	0.9793	0.9793
Resolution (Å)	40.6–2.304 (2.44–2.30)	43.20–2.01 (2.06–2.01)
Measured reflections (#)	101179	571503
Unique reflect. (#)	27791	57230
Data redundancy	3.6 (2.7)	13.6 (8.7)
Completeness (%)	99.0 (98.2)	99.9 (99.5)
R_sym_ (%)	4.0 (43)	6.0 (35.4)
I/σ_I_	22.21 (3.13)	14.6 (2.0)
**Refinement Statistics**		
Phasing	Molecular Replacement	Molecular Replacement
Molecules/AU	2 (no NCS applied)	2 (no NCS applied)
R_work_/R_free_ (%)	20.9/28.4	20.85/23.9
Free R test set size (/#%)	1357/5	2890/5
Number of protein atoms	3897	3912
Number of hetero-atoms	134	365
Mean B-factors (Å^2^) main chain	55.3	39.6
Mean B-factors (Å^2^) side chains and waters	58.8	45.8
Rmsd bond length (Å)	0.015	0.014
Rmsd bond angles (°)	1.551	1.407
Rmsd planes (Å)	0.008	0.007
Ramachandran most	95.75	97.98
Ramachandran additional	4.25	2.02

**Figure 2:**
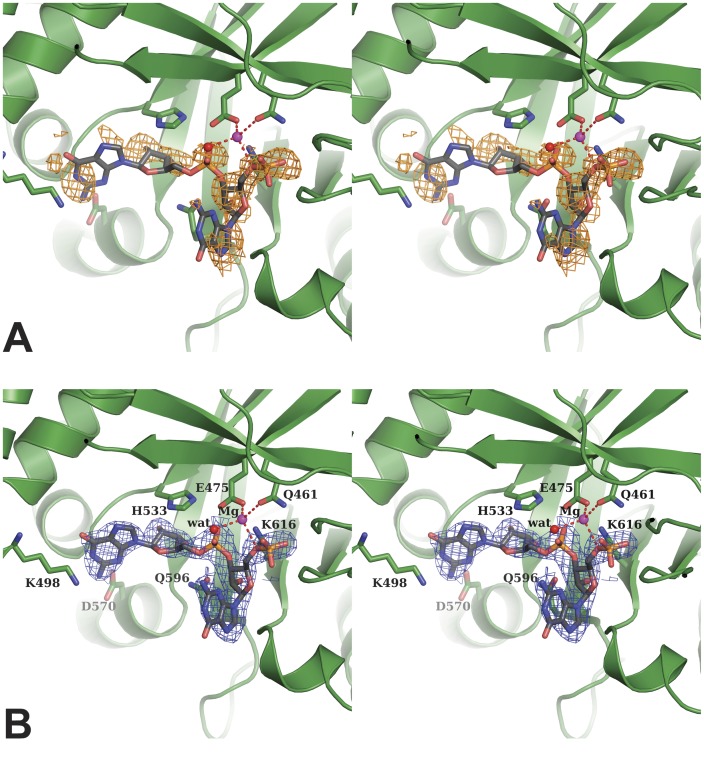
Stereo view of the active site of FimX-EAL domain. (A) The residual density Fo-Fc calculated after molecular replacement with no ligand in the model is contoured in orange at 3 σ level (A). The FimX backbone is represented as green cartoon. (B) Interpretation of the density as the bound 5′-pGpG, shown as black sticks. The magnesium ion is shown as a magenta sphere and the catalytic water involved in its coordination as a red sphere. Side-chains of residues involved in ligand binding are shown. The final electron density map 2Fo-Fc contoured at 1.2 σ level is traced around the ligand.

Residual density in the active site of both EAL domains of the asymmetric unit of the P4_3_2_1_2 crystal form leads us to place a molecule in the active site (Figure 2 A). Density maps were improved by simulated annealing omit maps. First attempts were carried out with c-di-GMP substrate but yielded very poor fit. Instead, the accommodation of 5′-pGpG, the reaction product, in the cavity was much better and acceptable. Density is clearly defined for 5′-pGpG phosphates and sugars but not for the two guanine bases (Figure 2 B). The structures of FimX-EAL crystallized with 5′-pGpG (P4_3_2_1_2 space group) will be further referred as the complex form, and that with no density in the active site (P2_1_2_1_2_1_ space group) as the apo form.

FimX-EAL displays a central barrel composed of 8 β-strands surrounded by 7 α-helices, with a αβ(β/α)_6_β secondary structure succession (Figure 1). This secondary structure topology has already been reported for EAL domains like YkuI [15] and is a variant of the triosephosphate isomerase (TIM) barrel fold. All the structure of EAL domains solved so far share this characteristic TIM-like barrel fold.

FimX-EAL displays very little variations within the asymmetric unit of both crystal forms with r.m.s. deviations of 0.47 Å over 249 superimposed Cα positions and 0.38 Å over 245 superimposed Cα positions in the complex and in the apo forms respectively. The structures of FimX-EAL complex and apo form are also very similar to each other with a r.m.s. deviation of 1.18 Å over 485 superimposed Cα positions.

Superposition of all known EAL domain structures was performed chain by chain. The core barrel of these two EAL domains is invariable. Deviation contributions arise from helices and loops. Interestingly, loop-8 is the only loop highly superimposable. The closest structure to ours is FimX-EAL solved previously (PDB code 3HV8) [21], then tdEAL (PDB code 2R6O) with a r.m.s deviation of 2.37 Å. *B. subtilis* YkuI (chain A) and our structure of *P. aeruginosa* FimX-EAL bound to 5′-pGpG (chain B) are the most divergent structures, with a r.m.s. deviation rising to 2.59 Å over 220 superimposed Cα positions.

### Apo FimX-EAL and FimX-EAL Bound to 5′-pGpG both Crystallize as Homodimers

**Figure 3:**
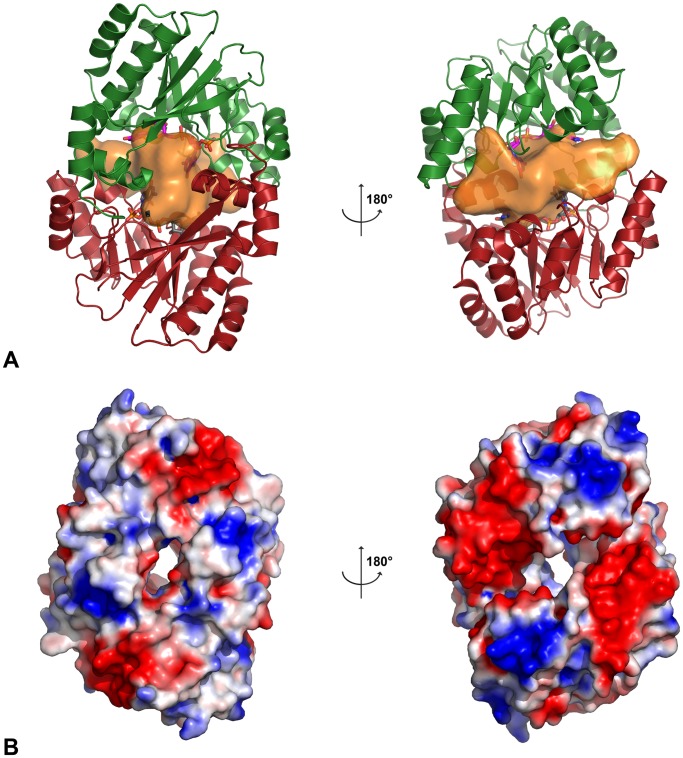
Representation of the cavity in the dimer of FimX-EAL bound to pGpG. (A) The cavity is presented as an orange surface inside the FimX-EAL dimer. It is connected to the outside by two channels of different size. Left and right views are rotated by 180°. The corresponding electrostatic surface is represented in (B). The edges of both channels are negatively charged and forbid the ligand to escape from the cavity.

FimX-EAL is monomeric in solution whether it is free or bound to c-di-GMP [21]. However in both our structures, two molecules are present per asymmetric units and form a homodimer (Figure 1 B). The surface of interaction is formed by the N- and C-terminal extremities and external α-helices and loops (Figure 1 and 3). In both cases, the α-helices and the loops responsible for the inter-monomer interactions are those surrounding the core barrel. In the apo form of the EAL domain, the secondary structures responsible for the interactions are α-helices 1, 2, 6, 7 and loops β2-α2, β7-α7 (previously described as loop 7 in TIM-barrel related literature). In the structure of FimX-EAL bound to 5′-pGpG, the interactions are mediated by contacts between α-helices 1, 2, 6, 7 and loops α1-β1, β5-α5. The surface of interaction measured by PISA is around 1410 Å^2^ with a score of 0.321 for the complex form and is 1430 Å^2^ with a score of 0.401 for the apo form. In both dimers, the computed scores for the interaction are relatively weak but there is however a large interface area due to hydrogen bonds and hydrophobic contacts. Similar homodimer was already described in the asymmetric unit of crystals in space group P4_1_2_1_2 and in the crystal lattice with a symmetry related molecule in space group P6_4_22 [21]. Thus, this homodimer exists in four crystal forms with different crystal packing.

Dimerization of FimX-EAL results in formation of a cavity with a volume of 4876 Å^3^ and 3609 Å^3^ for the complex and the apo forms respectively (Figure 3). In the complex form, two 5′-pGpG molecules are inside the cavity. They are in the same conformation and separated by a distance of 7.39 Å. They do not interact with each other and they are too far away from each other to allow bond formation or any reaction to proceed. It is interesting to note that the cavity is open to the solvent by two channels of 9.76 Å and 13.34 Å diameters (Figure 3 A). Electrostatic surface shows that the edges of these channels are negatively charged (Figure 3 B). The small diameters and the charges of the channels prevent 5′-pGpG molecules from escaping the cavity, trapping them inside the dimer like a bird in a cage.

Such a cage was previously described for a dimeric form of apo FimX-EAL [21]. The authors mentioned that if each EAL domain binds a c-di-GMP ligand, clashes would occur between the two c-di-GMP molecules. Our structure of FimX-EAL in complex with 5′-pGpG confirms that for the same reasons, FimX-EAL bound to c-di-GMP cannot exist as a dimer.

### Ligand Binding Site

**Figure 4:**
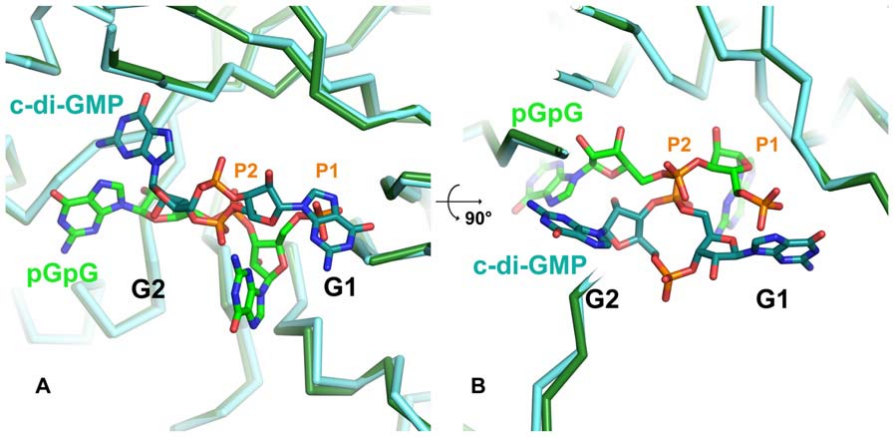
Comparison of binding of c-diGMP and 5′-pGpG. FimX-EAL in complex with 5′-pGpG (green) and c-di-GMP (cyan) (PDB code 3HV8) are superimposed. The phosphate groups of 5′-pGpG are numbered P1 and P2. The two views are rotated by 90° from each other.

**Figure 5:**
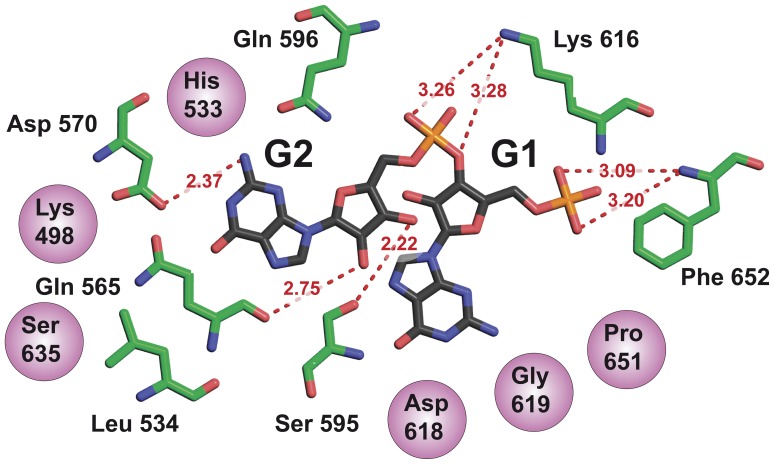
Schematics view of the interactions between FimX-EAL and 5′-pG-pG. 5′-pGpG is shown as black sticks. Residues of FimX-EAL involved in polar contacts are shown in green sticks and residues involved in hydrophobic interactions are shown as purple spheres. Polar contacts are shown as red dotted lines.

The ligand electron density map is well defined for phosphate and sugars but is incomplete for the two guanines (Figure 2). Several interactions are observed between the protein and the ribose-phosphate backbone by hydrogen bonds and hydrophobic contacts, showing that the ribose-phosphate is tightly trapped while the two bases orientation remains flexible. Residues involved in 5′-pGpG and Mg^2+^ binding mainly arise from the β-strands forming the barrel. 5′-pGpG binding residues are mainly located on strands β4, β5, β6 and loops 7 and 8, and magnesium-coordinating residues are on strands β1 and β2. The EAL domains core barrel is thus essential to ligand binding and consequently probably to the catalytic mechanism. Although our structure of FimX-EAL complexed with 5′-pGpG is highly superposable to that of FimX-EAL in complex with c-di-GMP (PDB code 3HV8), the overall position and conformation of the ligand in the active site are different in the two structures (Figure 4). However noteworthy, the non-hydrolyzed phosphate of 5′-pGpG (P2) lies in the same position as that of the c-di-GMP hydrolysable phosphate and share similar interactions with some amino acids, conversely to the hydrolyzed phosphate that is rocked 7.96 Å away from the position it occupies in c-di-GMP (Figure 4). To accommodate the differences of conformations, the residues involved in ligand binding are also different. In FimX-EAL in complex with c-di-GMP, Phe652 (loop 7) stacks on the guanine base. In FimX-EAL in complex with 5′-pGpG, it has rotated to make hydrogen bonds with the 5′-hydrolyzed phosphate (Figure 5). Gln596 (β5) has moved by 1.60 Å in order to allow hydrogen bonding between the amid group and 3′ hydroxyl of the ribose G2 of 5′-pGpG. Lys616 (β6) binds to the 2′ hydroxyl of the G1 ribose, Gln565 (β4) and Ser595 (β5) respectively to the 2′ hydroxyl and the 3′ hydroxyl of the ribose G2. Hydrophobic contacts concern principally base binding, with the exception of His533 (β3) that lies above to the second sugar (Figure 2).

### Magnesium Coordination

In our structure with the ligand, only one magnesium ion is present in the active site. The magnesium is coordinated in a tetrahedral state by Gln461 (β1) and Glu475 (β2) from the EAL motif (turned EVL in FimX), a water molecule and the 5′-phosphate from 5′-pGpG (Figure 2). The magnesium ion is essential for the PDE-A activity [14], [19]. A glutamate is involved in magnesium coordination in all known EAL domain structures, with the exception of FimX-EAL in complex with c-di-GMP (PDB code 3HV8) for which no magnesium ion at all is present in the structure. In our structure, the Mg^2+^ ion is shifted 5.9 Å apart from the position usually observed, but is still coordinated by Glu475 and one oxygen of the hydrolyzed phosphate.

### Phosphodiesterase Activity of FimX

**Figure 6:**
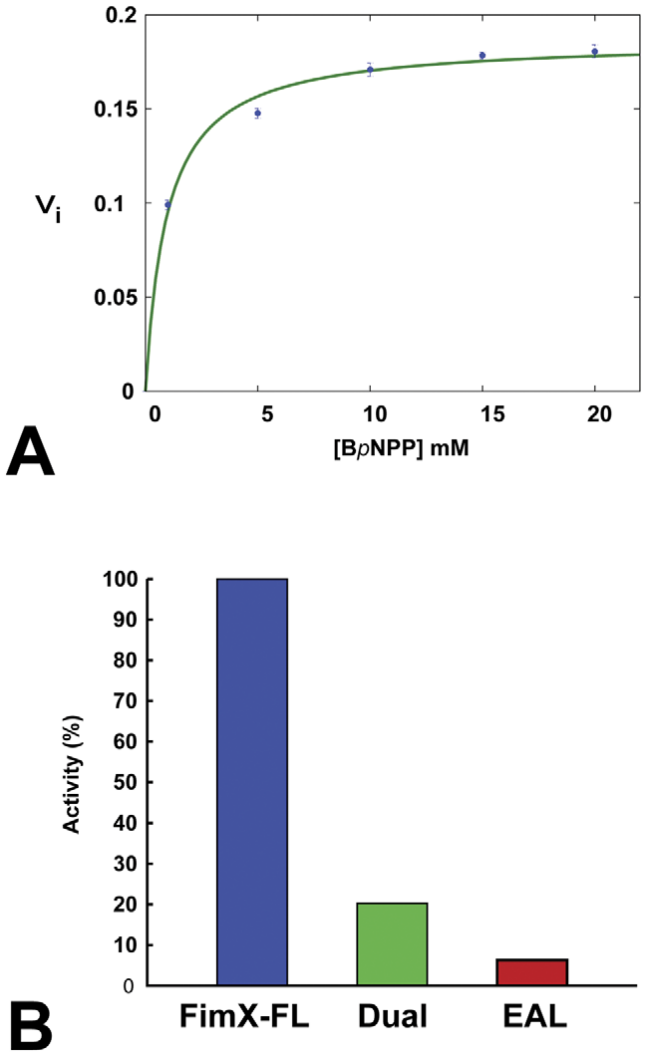
Enzymatic activity of hydrolysis of BpNPP by FimX. (A) Kinetics parameters of full-length FimX. Initial velocities are expressed in arbitrary units. (B) Relative activity of B*p*NPP hydrolysis after 9 hours of full-length FimX (FimX-FL), GGDEF-EAL domains (Dual) and EAL domain (EAL). Catalytic activities are normalised against that of FimX-FL. Dual has 18.1% activity and the EAL domain alone retains 6.6% activity.

In order to assess the phosphodiesterase activity of FimX and thus to validate the presence of 5′-pGpG in our crystallographic structure, we performed enzymatic assays using Bis (*p*-nitrophenyl) phosphate (B*p*NPP) as substrate in Mn^2+^ buffer. This compound was proved to be an alternative to c-di-GMP to measure PDE-A activities and was used with several EAL domains like HmsP [22], FimX [19], TBD1265 and SFV_3559 [16]. Though B*p*NPP isn’t the natural substrate of EAL domains, previous studies proved that a millimolar K_M_ for B*p*NPP corresponds to a nanomolar K_M_ for EAL domains for the natural substrate c-di-GMP. However the turnover kcat may display strong variation compared to the turnover for the natural substrate c-di-GMP [16]. The time course of the release of *p*-nitrophenol was measured for several hours as a function of the B*p*NPP concentration (1 mM, 5 mM, 10 mM, 15 mM and 20 mM) and initial rates were determined for each concentration. Full-length FimX displays a K_M_ of 1 mM and a kcat of 6.4.10^−4 ^s^−1^ (Figure 6 A). K_M_ measured for full-length FimX is lower to the one measured for TBD1265 and SFV_3559 (20 mM and 14 mM respectively). In contrary, kcat is weaker than the one measured for TBD1265 and SFV_3559 EAL domains (0.06 s^−1^ and 8 s^−1^ respectively). The catalytic efficiency kcat/K_M_ of full-length-FimX is 10-fold lower than the one of TBD1265. These experiments prove that FimX full-length displays a strong affinity for its substrates but a very weak turnover considering its K_M_ and kcat. FimX has a low activity with B*p*NPP as substrate. This low phosphodiesterase activity is in agreement with what was already described [19].

We performed a relative activity assay over nine hours in order to determine whether isolated GGDEF-EAL and EAL domains of FimX also display enzymatic activities. Our results indicate that GGDEF-EAL domains activity is five-fold weaker than FimX full-length activity. Phosphodiesterase activity of EAL domain is around three-fold weaker than GGDEF-EAL domain activity and fifteenth-fold weaker than FimX full-length activity (Figure 6 B). The observation of a very weak phosphodiesterase activity validates the formation of 5′-pGpG during the time course of FimX-EAL crystallization.

### c-di-GMP and 5′-pGpG both Bind to FimX-EAL in Solution

**Figure 7:**
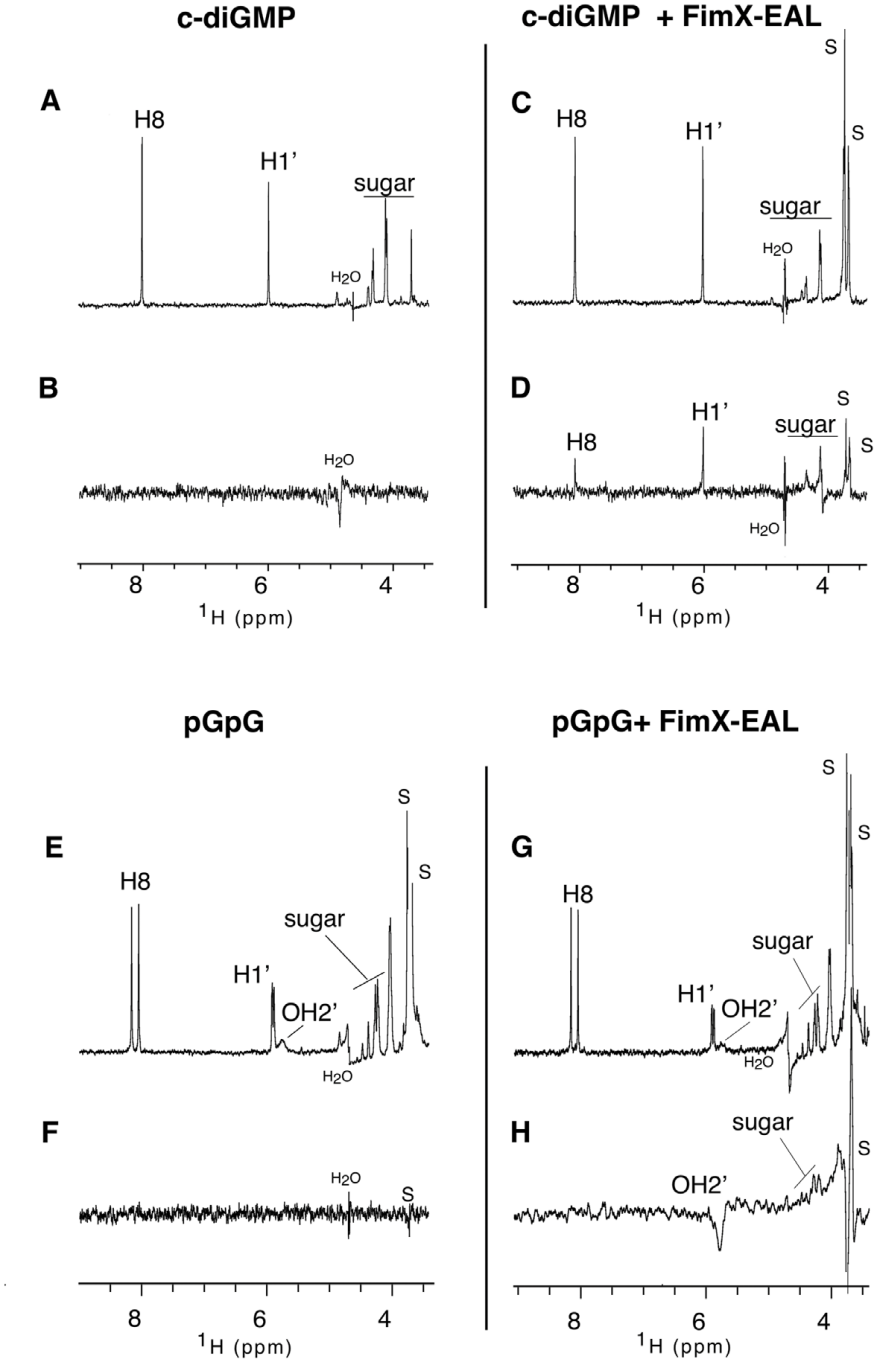
Reverse NOE-pumping experiments. Expanded 1D NMR spectra recorded in D_2_O at 600 MHz and 308 K, pH 6.5 of c-di-GMP and 5′-pGpG alone (respectively left panels A-B and E-F) and with FimX-EAL (right panels C-D and G-H). Panels A, C, E and G: reference 1D-esgp experiments. Panels B, D, F and H: RNP experiments. (S = signals arising from buffer).

We wanted to probe c-di-GMP and 5′-pGpG binding to the FimX-EAL domain in solution and identify which part of the ligand is involved in the interaction. We have thus carried out a series of reverse NOE-pumping (RNP) NMR experiments [23] in D_2_O (H_2_O contaminated) buffer at 308 K and pH 6.5, for which only small amounts of unlabeled protein and an excess of ligand are required (Figure 7). The H_2_O contamination allows potential observation of magnetization transfers to sugar hydroxyl protons. No magnesium was added to the NMR buffer to prevent any enzymatic reaction or degradation of the ligands during spectra recording. The RNP experiment contains two blocks, a T2 relaxation filter selecting only the spin systems of the free ligand and a cross-polarization module allowing intermolecular NOE magnetization transfer between the protons of the bound-ligand and those of the protein. Two spectra are recorded sequentially with inverted modules order and subtracted. In the end, the difference spectrum only displays the peaks arising from protons of the ligand that are within a few Å from those of the protein and thus susceptible to interact with the protein (Figures 7 D and G). As controls, in absence of protein, no signal is observed (Figures 7 B and F). The NOE mixing time can be varied to probe short to larger intermolecular distances. When c-di-GMP is mixed FimX-EAL, the RNP experiment discloses two transfers, one to a G H8 (ppm) and the other to a G H1’ (ppm) (Figure 7 D). This shows that, the recognition of c-di-GMP involves at least one base and one sugar of the ligand. This is consistent with the crystallographic structure, which discloses that one guanine is stacked on a phenylalanine of the EAL domain [21]. Conversely, in the same experimental conditions (NOE mixing time of 400 ms), no transfers are observed when 5′-pGpG is mixed with FimX-EAL. However, longer mixing times (here 700 ms) allow observation of a strong transfer to one OH2’ sugar hydroxyl (negative signal at 5.7 ppm) and of a weaker transfer to H5′/5″ (positive peaks at about 4.5 ppm), the observation of the latter transfer is slightly impeded by traces of the buffer used by Dharmacon to purify 5′-pGpG that we were not able to remove without loss of considerable amounts of the product). However, no transfer to G H8 and H1′ protons could be recorded, even when temperature was lowered to 298 K (not shown) to increase the affinity. Our RNP experiment shows that in solution and at relatively high temperature, even in the absence of magnesium, FimX-EAL can bind to the 5′-pGpG albeit much weaker than to c-di-GMP. The fact that no transfers to GH8 and H1′ are observed, are consistently with our crystallographic results which show that none of the guanine aromatic bases are involved in the binding. The specific and reproducible absence of some magnetization transfers also rules out magnetization transfers to non-specifically bounded 5′-pGpG molecules.

## Discussion

### FimX-EAL is Able to Crystallize as Homodimer

We have crystallized the FimX-EAL in its apo form and in complex with 5′-pGpG as a homodimer. It was previously shown that FimX-EAL bound to c-di-GMP crystallized as a monomer because dimerization would cause a large number of ligand-ligand clashes in the binding pocket. Dimerization of apo FimX-EAL was thought to prevent c-di-GMP binding [21]. We observed here that FimX-EAL crystallizes in two new crystal forms as a dimer with a large interface area and the involvement of several polar residues of each monomer. The propensity of FimX-EAL domains to dimerize is emphasized by the observation that they can crystallize as homodimers in different space groups even without ligand. Though the large contact area between the two monomers and the number of residues involved in electrostatic bonding at the interface are compatible with the formation of FimX-EAL dimers in solution, this dimer was never observed *in vitro*. Gel filtration and SAXS experiments on FimX-EAL with or without 5′-pGpG could not reveal the formation of a dimer in solution (data not shown). The dimerization interface involves mainly N- and C-terminal extremities residues, and in less extent helices α2, α6 and α7 helices. The N- and C- terminal extremities may be more flexible in solution than in the crystal context. Structural rearrangements of the flexible N- and C-terminus may be necessary to form the oligomer and crystal packing could stabilize these conformations. Because the residues involved in the interface are mostly polar, burying polar residues during dimerization does not have a large contribution to the entropic effect and will not stabilize the dimer.

The oligomeric state of EAL domains and full-length proteins containing an EAL domain is very variable and depends on the ligand bound to the domain. Several EAL domains, like YkuI, BlrP1, YcgF and tdEAL have been reported to also form EAL-EAL mediated homodimers in crystals when they are bound to c-di-GMP and for some of them in the apo form. The existence of homodimeric forms was confirmed in solution by analytical ultracentrifugation, size exclusion chromatography and dynamic light scattering [15], [16], [24]. RocR from *P. aeruginosa* is tetrameric in solution [25]. Former X-ray scattering experiments also show that both apo FimX-EAL and apo FimX-EAL-GGDEF are monomeric in solution. Only full-length apo FimX is able to dimerize, most probably through its N-terminal domain [21]. These results of X-ray scattering experiments were the same whether FimX or its constructs were complexed or not with c-di-GMP. As FimX is a long and multi-domain protein, we cannot exclude the possibility of several interfaces promoting dimerization, outside of the N-terminal domains suggested by the SAXS experiments on the apo protein [21].

### Product Binding

FimX has a strong affinity for c-di-GMP according to the Kd (about 120 nM) computed from ITC experiments [21], [26]. Our RNP NMR experiments confirm that FimX-EAL binds c-di-GMP in solution, and, in much less extent, 5′-pGpG, the product of the hydrolysis of c-di-GMP. The 5′-pGpG was formed during crystallization by either the very weak phosphodiesterase activity of FimX-EAL or by spontaneous hydrolysis of c-di-GMP. However, the product formed was trapped in the FimX-EAL dimer formed in the crystals. The crystallographic bias allowed us to obtain the structure of FimX-EAL in complex with reaction product.

**Figure 8:**
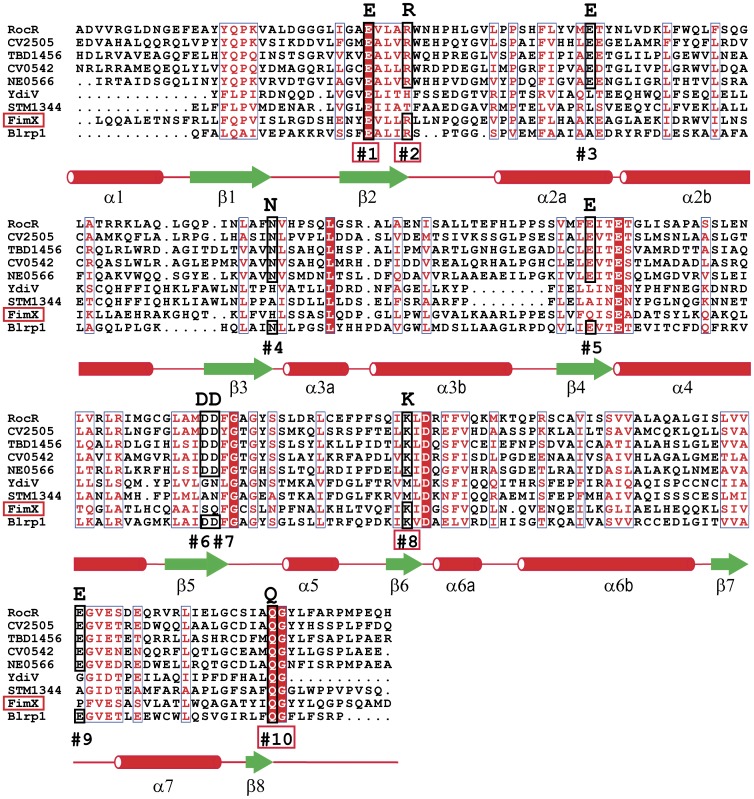
Sequence alignments of EAL domains from various proteins. Alignement of EAL domains was done with ClustalW. Conserved residues important for the PDE-A activity are numbered #1 to #10. Residues conserved in FimX-EAL are boxed in red. The secondary structure is indicated for FimX-EAL structure.

Interestingly, in the complexes, the 5′-pGpG conformation is very different from that of the c-di-GMP preventing clashes between the two ligands inside the dimeric catalytic pocket. The only position to be conserved is that on the non-hydrolyzed phosphate in 5′-pGpG which occupies the positions of the target phosphate of c-di-GMP in the hydrolysis reaction. 5′-pGpG is thus in position for a potential second round of hydrolysis. Neither the guanines nor the sugars of 5′-pGpG are superimposed on their counterpart in c-di-GMP. Conversely to c-di-GMP, and according to both our crystallographic structure and NMR data, none of the two guanines seem involved in the 5′-pGpG recognition. We performed a sequence alignment of FimX with other EAL domains in order to define which of the ten conserved residues identified as essentials for the catalytic activity [16] are retained (Figure 8). We show that FimX-EAL domain has four of them at positions 1, 2, 8 and 10. Glu in position 1 is part of the EAL motif and was defined as an essential residue for magnesium coordination. This is confirmed by our structure where Glu475 coordinates the magnesium ion. Residue number 2 is conserved in FimX-EAL and corresponds to Arg479, but it is neither involved in magnesium coordination nor in 5′-pGpG binding. Residue in positions 8 corresponds to Lys616 and is involved in substrate binding. Residue 10 (here Glu671) has no known function and is not involved here in ligand binding.

The number or coordinated magnesium ions in the catalytic site of the EAL domains varies amongst the structures: in BlrP1 and tdEAL, two magnesium ions are present [16], [24], in YkuI, only one magnesium ion is present in the catalytic site [15]. The coordination state of magnesium in EAL also presents some variability: the two metal ions in BlrP1 are hexa-coordinated [24], but in a magnesium of tdEAL and our structure of FimX-EAL bound to 5′-pGpG, magnesium ions are tetra-coordinated. The coordination of magnesium in our structure corresponds to the tetra-coordinated calcium found in the structure of *B. subtilis* YkuI bound to c-di-GMP [15]. We confirm the essential role of one glutamate from the EAL motif, in this coordination as suggested by previous bioinformatics and sequence alignment studies. Our structure supports a previous model of c-di-GMP hydrolysis in which Glu475 would act as a general base catalyst, activating Mg^2+^. After the hydrolysis of the c-di-GMP, the Mg^2+^ is not located at the conserved position observed in other EAL domains. It remains coordinated by a water molecule, by one of the two oxygens of the hydrolysed phosphate, by a carboxylic oxygen from Glu475 of the EAL motif and by the backbone carbonyl of Gln461. This network of interactions supports a catalytic mechanism in which a water molecule activated by the cation would perform a nucleophilic attack on one of the cyclic phosphates and hydrolyse the c-di-GMP as proposed for YkuI and BlrP1. According to this model, during the reaction, the hydrolysed phosphate would move away from its original position, dragging the Mg^2+^, and induce conformation changes of the whole hydrolyzed ligand backbone, resulting also in a change of the positions of the bases. The fact that only Glu475, a water molecule and one magnesium ion are essential to carry out the hydrolysis of the c-di-GMP, could explain why the overall structure of the FimX-EAL domain doesn’t undergo major structural rearrangements during the hydrolysis. Interestingly, the position of the hydrolysed phosphate is highly superimposable to the equivalent position *Leishmania mexicana* TIM in the complex with 2-(N-formyl-N-hydroxy)-aminoethyl phosphonate (PDB code 1IF2), suggesting the existence of a conserved phosphate-binding motif involving the C-terminal loops of TIM and EAL domains. Our results also suggest that the exact number of cations involved in the catalysis may not be fixed amongst EAL catalytic pockets.

We can’t exclude the possibility that other proteins could interact with FimX. Our results coupled to previous ones, allow us to assess that there is no major structural rearrangement on EAL domain of FimX whether it is in the apo- form or bound to 5′-pGpG or c-di-GMP. Recent results obtained by mass spectrometry on full-length FimX indicate that the binding of c-di-GMP induces long-range conformation changes and that this allosteric regulation could modulate the binding of a partner protein [26]. Interestingly, NMR and two-hybrid assays in *Xanthomonas* proved that FimX homolog is able to bind PilZ, a major protein in type IV pili biogenesis, via its EAL domain [18]. It would be interesting to determine what is the effect of 5′-pGpG binding on the overall structure of full-length FimX in solution and what would be the consequences on oligomerization of the protein.

As a conclusion, we bring here the first high-resolution structure of an EAL domain complexed with 5′-pGpG. This is the first time this reaction product of c-di-GMP hydrolysis is trapped within its ligand binding pocket, like a bird in a cage. The analysis of the new network of interactions between the protein, the ligand, a water molecule and the metallic cation support the model of hydrolysis of c-di-GMP by EAL domains proposed previously.

## Materials and Methods

### Cloning

The full-length FimX protein (amino acids 1–691), the GGDEF-EAL domains (amino acids 256–691) and the EAL domain (amino acids 439–691) were amplified by PCR from *Pseudomonas aeruginosa* PA01 genomic DNA and cloned into the pET-15b vector (Novagen). The complete FimX was amplified using forward primer 5′-CACAACCATATGGCCATCGAAAAGAAAACCATC and reverse primer 5′-ATACGGGATCCTCATTCGTCTCCCGAGGAGAAGTCGTA using HotStart HiFi Taq DNA polymerase (Qiagen). The PCR product was digested with NdeI and BamHI (New England Biolabs) and cloned into NdeI and BamHI restriction sites. Similarly, GGDEF-EAL domain was amplified using forward primer 5′-GGCGAGCATATGAACGCCGAGCTGGAAGAAAAGCTC and reverse primer 5′-TGCATACTCGAGTCATTCGTCTCCCGAGGAGAAGTC and cloned into NdeI and XhoI sites. EAL domain was amplified with forward primer 5′-GCCGCCCATATGCAGCGCGGCGACGTCATCGCGATC and reverse primer 5′-TGCATACTCGAGTCATTCGTCTCCCGAGGAGAAGTC and cloned into NdeI and XhoI sites.

*Escherichia coli* BL21 strains were transformed with the recombinant plasmids for protein expression. All the N-terminal his-tagged proteins were purified using the same protocol. Bacteria were grown at 37°C up to OD 0.6. Recombinant protein production was induced by addition of 0.5 mM IPTG. Bacteria were then grown for a further 3 hours at 37°C. Cells were harvested by centrifugation at 4000 g for 20 minutes. The bacteria pellet was re-suspended in lysis buffer (NaH_2_P0_4_ 50 mM pH 8.5, NaCl 300 mM, Imidazole 20 mM, lysozyme 0.5 mg/ml, PMSF 0.5 mM, bensonase 100 U) and sonicated for 3 minutes. The sonicate was clarified by centrifugation at 10000 g for 30 minutes. The supernatant was loaded onto a Ni^2+^-NTA column (Qiagen). The column was extensively washed with lysis buffer and the protein was eluted with NaH_2_PO_4_ 50 mM, NaCl 300 mM, Imidazole 500 mM. The protein was then purified by size exclusion chromatography on a HiLoad Superdex 200 16/60 (GE Healthcare) equilibrated in Tris-HCl 50 mM, NaCl 300 mM at a flow rate of 1 ml/min. Fractions of interest were analysed on SDS-PAGE and pooled. Proteins were further concentrated on centricon (Amicon).

### Enzymatic Assays

Phosphodiesterase activities of FimX-EAL, FimX GGDEF-EAL and full-length FimX was measured with Bis(*p*-nitrophenyl) phosphate (B*p*NPP) (Sigma-Aldrich) as substrate. B*p*NPP was diluted in 200 mM NaCl, 50 mM Tris-HCl pH 8.5 and 1 mM MnCl_2_ to the final concentrations of 1 mM, 5 mM, 10 mM, 15 mM and 20 mM [22]. Standard assays were performed at 37°C for 5 hours and 30 minutes and the release of the reaction product (*p*-nitrophenol) was monitored and quantified at 410 nm at different times. Absorption curves were plotted against time and kinetics parameters extracted from Hanes-Woolf linearization [27].

For comparison of catalytic activities of FimX full-length, GGDEF-EAL and EAL domains, enzymatic assays were performed in reaction buffer 200 mM NaCl, 50 mM Tris-HCl pH 8.5, 1 mM MnCl_2_ and B*p*NPP at a final concentration of 5 mM. Reaction mixture was incubated at 37°C for 9 hours and absorption was measured at 410 nm.

### Crystallography

FimX-EAL (0.5 mM) alone or in equimolar complex with MgCl_2_ and c-di-GMP (Biolog Life Science Institute, Gmbh) was used for crystallization. Crystallisation conditions were screened with the JCSG kit (Qiagen) on a CyBIO crystallisation robot. FimX-EAL alone crystallized in Na-Acetate 0.1 M pH 4.5, K_2_HPO_4_ 0.4 M, NaH_2_PO_4_ 1.2 M. Crystals of FimX-EAL complex were obtained by vapour diffusion in Hepes 0.1 M pH 7.5, NaH_2_PO_4_ 0.8 M and K_2_HPO_4_ 0.8 M. Crystals were cryoprotected with a brief soaking in a solution containing crystallisation buffer and 30% (v/v) glycerol. Diffraction data were collected on Proxima 1 beamline at SOLEIL synchrotron, processed with XDS [28]. Molecular replacement was done using Balbes [29] with the tdEAL structure (PDB code 2R6O) as template. The molecular replacement solution structure was automatically rebuilt using Phenix [30]. Manual reconstruction and ligand fitting were achieved using Coot [31]. Refinement was performed with Phenix. Structural analysis of the surface and interface was done with Protein interfaces, surfaces and assemblies service PISA at European Bioinformatics Institute [32]. Figures for molecular structure description were done with PyMOL [33].

### NMR

NMR spectra were recorded on a 600 MHz Bruker Avance spectrometer equipped with a TBI probe and X,Y,Z-gradients and samples loaded in a 5 mM Shigemi tubes. The ligands (c-di-GMP, BioLog; 5′pGpG, Dharmacon) were dissolved in water to a concentration of 0.5 mM and the pH adjusted to 6.5 by the addition under stirring of small amounts of NaOH 0.1 N. The solutions were then lyophilized and re-suspended in the same volume of D_2_O. *P. aeruginosa* FimX-EAL was dissolved from a concentrated aqueous stock solution by the 0.5 mM ligand NMR solution to a final concentration of 30 uM. 1D proton spectra were recorded using gradient sculpting for water suppression. Reverse NOE-pumping experiments [23] were acquired with a 50 ms CMPG sequence and 400 and 700 ms NOE mixing times. Data were processed using Topspin 2.0 (Bruker).

### Accession Numbers

Coordinates and structure factors have been deposited in the Protein Data Bank with accession number 4AG0 (FimX-EAL apo) and 4AFY. (FimX-EAL in complex with 5′-pGpG).

## Supporting Information

Figure S1 SAXS experiment on FimX EAL in Tris-HCl 50 mM, NaCl 200 mM, MgCl_2_ 1 mM, pH 7.5. (A) Absorbance of the elution profile is recorded at 260 nm and 280 nm. Scattering data corresponding to the first peak (16.68 min) is shown (B) with corresponding Guinier analysis (C). Estimated molecular weight is 33362 Da (expected 30245 Da for FimX EAL monomer)
(TIF)

Figure S2 SAXS experiment on FimX EAL mixed with 5′-pGpG (in 100 fold excess) in Tris-HCl 50 mM, NaCl 200 mM, MgCl_2_ 1 mM, pH 7.5. (A) Absorbance of the elution profile is recorded at 260 nm and 280 nm. Scattering data corresponding to the first peak (16.738 min) is shown (B) with corresponding Guinier analysis (C). Estimated molecular weight is 28336 Da (expected 30245 Da for FimX EAL monomer). The peak at 19.31 min is free 5′-pGpG.
(TIF)

Materials and Methods S1 Materials and methods for SAXS experiments.
(DOC)
