# Supplementary figures and images for: Crystal Structure of an EAL Domain in Complex with Reaction Product 5′-pGpG
Source: PLoS One. 2012 Dec 20;7(12):e52424. doi: 10.1371/journal.pone.0052424 (PMC3527489; doi:10.1371/journal.pone.0052424)

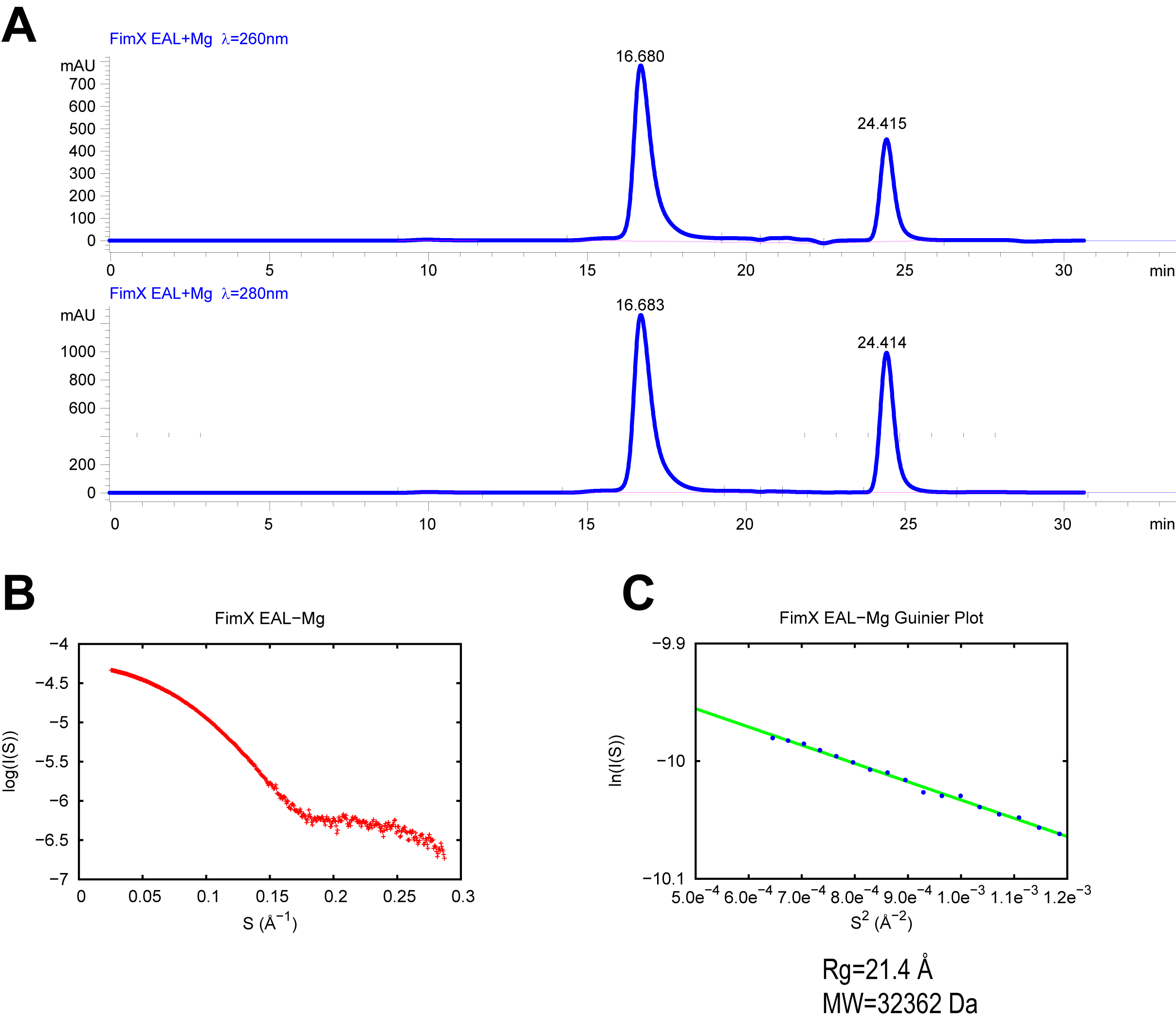

Supplement: Figure S1 — SAXS experiment on FimX EAL in Tris-HCl 50 mM, NaCl 200 mM, MgCl2 1 mM, pH 7.5. (A) Absorbance of the elution profile is recorded at 260 nm and 280 nm. Scattering data corresponding to the first peak (16.68 min) is shown (B) with corresponding Guinier analysis (C). Estimated molecular weight is 33362 Da (expected 30245 Da for FimX EAL monomer) (TIF) [file pone.0052424.s001.tif]

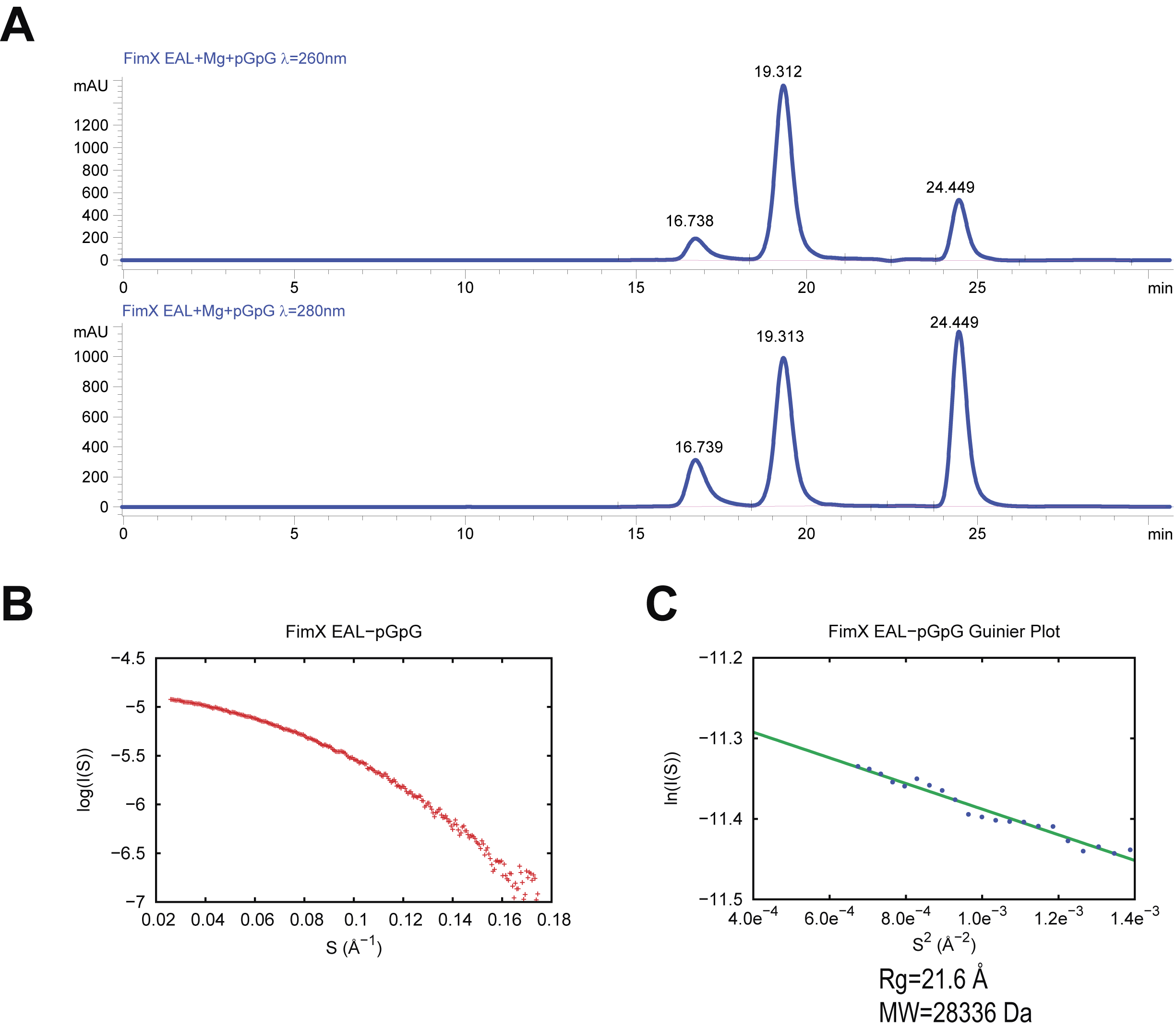

Supplement: Figure S2 — SAXS experiment on FimX EAL mixed with 5′-pGpG (in 100 fold excess) in Tris-HCl 50 mM, NaCl 200 mM, MgCl2 1 mM, pH 7.5. (A) Absorbance of the elution profile is recorded at 260 nm and 280 nm. Scattering data corresponding to the first peak (16.738 min) is shown (B) with corresponding Guinier analysis (C). Estimated molecular weight is 28336 Da (expected 30245 Da for FimX EAL monomer). The peak at 19.31 min is free 5′-pGpG. (TIF) [file pone.0052424.s002.tif]
