# Supplementary material for: Crystal Structure of an EAL Domain in Complex with Reaction Product 5′-pGpG
Source: PLoS One. 2012 Dec 20;7(12):e52424. doi: 10.1371/journal.pone.0052424 (PMC3527489; doi:10.1371/journal.pone.0052424)
Supplement: Materials and Methods S1 — Materials and methods for SAXS experiments. (DOC) [file pone.0052424.s003.doc]

Materials and methods

SAXS experiment

SAXS data were collected on the beamline SWING at the Soleil Synchrotron (Saint-Aubin, France) at an electron energy of 13.32keV. Protein samples were centrifuged at 13,200 x g for 10 min before data acquisition and concentration was measured with Nanodrop spectrophotometer (Thermo). Samples were loaded on a Agilent SEC-3 size-exclusion chromatography column connected to an Agilent HPLC system at a flow rate of 200μL.min^-1^. One hundred frames, with exposure times of 1 seconds per frame, were collected at 20°C in the flow-cell connected to HPLC system. Buffer background scattering was collected using gel filtration buffers (Tris-HCl 50mM, NaCl 200mM, pH 7.5). Peak analysis, background subtraction, averaging, and scaling were carried out using the program FOXTROT available on the beamline. Further processing and data analysis was done using the programs PRIMUS and the ATSAS suite. Only scattering data with S_max_*R_g_<1.3, computed from Guinier plots at low angle regions, was considered for further analysis.
